# Supplementary figures and images for: Artificial light at night alters life history in a nocturnal orb-web spider
Source: PeerJ. 2018 Oct 9;6:e5599. doi: 10.7717/peerj.5599 (PMC6183507; doi:10.7717/peerj.5599)

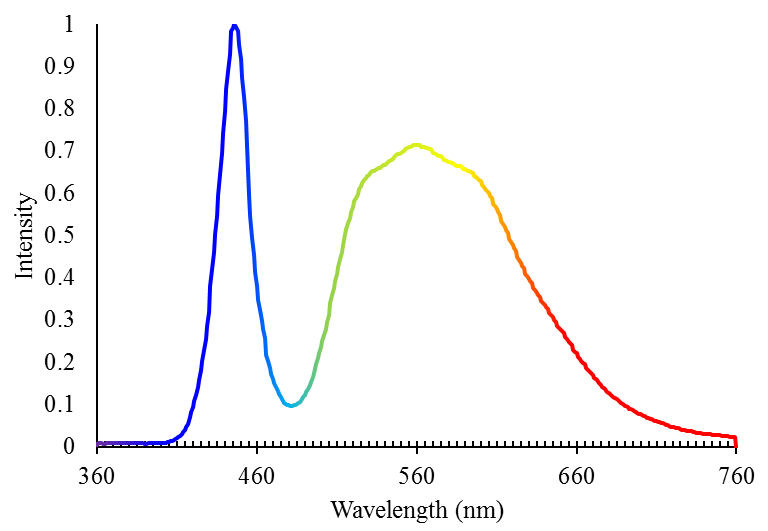

Supplement: Supplemental Information 1 — Spectral composition of the cool white LED lights (12V DC cool white LED strip lighting supplied by World of Thought, Victoria, Australia) used during rearing experiment. The blue peak wavelength is 445 nm. [file peerj-06-5599-s001.png]
